# CellML 2.0

**DOI:** 10.1515/jib-2020-0021

**Published:** 2020-07-24

**Authors:** Michael Clerx, Michael T. Cooling, Jonathan Cooper, Alan Garny, Keri Moyle, David P. Nickerson, Poul M. F. Nielsen, Hugh Sorby

**Affiliations:** University of Oxford, Oxford, UK; University of Auckland, Auckland, New Zealand; University College London, London, UK

**Keywords:** computational physiology, modularity, physiome project, reproducibility, reusability

## Abstract

We present here CellML 2.0, an XML-based language for describing and exchanging mathematical models of physiological systems. MathML embedded in CellML documents is used to define the underlying mathematics of models. Models consist of a network of reusable components, each with variables and equations giving relationships between those variables. Models may import other models to create systems of increasing complexity. CellML 2.0 is defined by the normative specification presented here, prescribing the CellML syntax and the rules by which it should be used. The normative specification is intended primarily for the developers of software tools which directly consume CellML syntax. Users of CellML models may prefer to browse the informative rendering of the specification (https://cellml.org/specifications/cellml_2.0/) which extends the normative specification with explanations of the rules combined with examples of their usage.

***Corresponding author: David P. Nickerson**, University of Auckland, Auckland, New Zealand, E-mail: d.nickerson@auckland.ac.nz. https://orcid.org/0000-0003-4667-9779
**Michael Clerx:** University of Oxford, Oxford, UK. https://orcid.org/0000-0003-4062-3061
**Michael T. Cooling, Alan Garny, Keri Moyle, Poul M. F. Nielsen and Hugh Sorby:** University of Auckland, Auckland, New Zealand. https://orcid.org/0000-0002-3417-0177 (M.T. Cooling). https://orcid.org/0000-0001-7606-5888 (A. Garny). https://orcid.org/0000-0002-7178-2631 (K. Moyle). https://orcid.org/0000-0002-4704-0179 (P.M.F. Nielsen). https://orcid.org/0000-0001-8991-4703 (H. Sorby)
**Jonathan Cooper:** University College London, London, UK. https://orcid.org/0000-0001-6009-3542

## CONTENTS

**1 Definitions** **2**
- 1.1 Terminology . . . 2
- 1.2 CellML information sets . . . 3
- 1.3 Data representation formats in CellML . . . 5

**2 Element information items** **6**
- 2.1 The model element . . . 6
- 2.2 The import element . . . 7
- 2.3 The import units element . . . 8
- 2.4 The import component element . . . 9
- 2.5 The units element . . . 10
- 2.6 The unit element . . . 11
- 2.7 The component element . . . 12
- 2.8 The variable element . . . 13
- 2.9 The reset element . . . 14
- 2.10 The test_value element . . . 15
- 2.11 The reset_value element . . . 16
- 2.12 The math element . . . 17
- 2.13 The encapsulation element . . . 18
- 2.14 The component_ref element . . . 19
- 2.15 The connection element . . . 20
- 2.16 The map_variables element . . . 21

**3 Interpretation** **22**
- 3.1 Interpretation of import elements . . . 22
- 3.2 Units references . . . 23
- 3.3 Interpretation of units elements . . . 24
- 3.4 Component references . . . 26
- 3.5 Variable references . . . 27
- 3.6 Interpretation of initial_value attributes . . . 28
- 3.7 Effect of units on a variable . . . 29
- 3.8 Interpretation of math elements . . . 30
- 3.9 Interpretation of encapsulation elements . . . 31
- 3.10 Interpretation of map_variables elements . . . 32
- 3.11 Interpretation of reset elements . . . 34

**4 References** **35**

This document is the normative version of the CellML Specification, defining the CellML syntax and the rules by which it should be used. It is intended primarily for the developers of software tools which directly consume CellML syntax.

Users of CellML models may prefer the informative version of the CellML Specification, which is available at https://cellml.org/specifications/cellml_2.0/.

The official version of the CellML Normative Specification is available at https://cellml.org/specifications/cellml_2.0/cellml_2_0_normative_specification.pdf.

**Authors:**

Michael Clerx, Michael T. Cooling, Jonathan Cooper, Alan Garny, Keri Moyle, David P. Nickerson, Poul Nielsen, and Hugh Sorby.

**Contributors:**

Koray Atalag, David Brooks, Edmund J. Crampin, Jesús Carro Fernández, Peter J. Hunter, Gary R. Mirams, and Maxwell L. Neal.

The authors also wish to acknowledge the significant contribution of the (discontinued) CellML 1.2 draft specification, much of the text of which was incorporated into this specification, although the semantics have changed considerably. The CellML 1.2 draft specification was itself the result of a collaborative effort by a number of researchers during 2008-2011:

Andrew K. Miller (who wrote the text reused here), Randall Britten, Jonathan Cooper, Alan Garny, Peter J. Hunter, Justin Marsh, Poul Nielsen, David P. Nickerson, and Hugh Sorby.

**Contact:** editors@cellml.org

CHAPTER

## ONE

## DEFINITIONS

### 1.1 Terminology

The keywords "MUST", "MUST NOT", "SHALL", "SHALL NOT", and "MAY" in this document are to be interpreted as described in RFC 2119 [1].

The key phrase "information item", as well as any specific type of information item such as an "element information item", are to be interpreted as described in XML Information Set [2].

**CellML infoset:** An XML information set containing a hierarchy of information items conforming to the rules described in this document. In this specification, such infosets are assumed to be CellML 2.0 infosets.

**CellML model:** A mathematical model represented by a hierarchy of one or more CellML infosets, according to the rules described in this document. In this specification, the topmost CellML infoset in a hierarchy is referred to as the top-level CellML infoset.

**Namespace:** An XML namespace, as defined in Namespaces in XML 1.1 [3].

**CellML namespace:** The CellML 2.0 namespace.

**CellML 2.0 namespace:** The namespace http://www.cellml.org/cellml/2.0#.

**MathML namespace:** The namespace http://www.w3.org/1998/Math/MathML.

**CellML information item:** Any information item in the CellML namespace.

**Basic Latin alphabetical character:** A Unicode [4] character in the range U+0041 to U+005A or in the range U+0061 to U+007A.

**Digit:** A Unicode character in the range U+0030 to U+0039.

**Basic Latin alphanumerical character:** A Unicode character which is either a Basic Latin alphabetical character or a digit.

**Basic Latin underscore:** The Unicode character U+005F.

**Basic Latin plus:** The Unicode character U+002B.

**Basic Latin minus:** The Unicode character U+002D.

**Basic Latin full stop:** The Unicode character U+002E.

**Whitespace character:** Any one of the Unicode characters U+0009, U+000A, U+000D, or U+0020.

### 1.2 CellML information sets

#### 1.2.1 CellML and XML

1. Every CellML infoset SHALL be represented in an XML information set which conforms with the well-formedness requirements of XML 1.1 [5].
2. In this document, the remaining provisions relating to CellML infosets SHALL be interpreted as additional constraints on the XML information set represented by a CellML infoset.

#### 1.2.2 Specific information items

1. For the purposes of this specification, a specific information item is one of the following (see https://www.w3.org/TR/2004/REC-xml-infoset-20040204/ for definitions):
    1. A document information item;
    2. An element information item;
    3. An attribute information item;
    4. A processing instruction information item;
    5. An unexpanded entity reference information item;
    6. A document type declaration information item;
    7. An unparsed entity information item; or
    8. A notational information item.
2. Specific information items MUST NOT appear in a CellML infoset except where explicitly allowed by this specification, or where allowed by a normative specification referenced by this specification.
3. The order in which specific information items appear, as children of an element information item defined in this specification, SHALL NOT affect the semantic interpretation of the CellML model.

#### 1.2.3 Non-specific information items

1. For the purposes of this specification, a non-specific information item is one of the following (see https://www.w3.org/TR/2004/REC-xml-infoset-20040204/ for definitions):
    1. A comment information item;
    2. A namespace information item; or
    3. A character information item.
2. An element information item in the CellML namespace MUST NOT contain any character information items, except for whitespace characters.
3. Two CellML infosets SHALL be deemed semantically equivalent if one can be transformed into the other by making zero or more of the following changes:
    1. Adding, removing, and/or modifying comment information items.
    2. Changing (inserting, removing, and/or modifying) one or more namespace information items, and/or modifying the prefix of one or more information items, without changing the namespace that any information item is in.
    3. The following paragraph applies to character information items which are the direct child of an element information item in the CellML namespace, or in the MathML namespace: inserting or removing character information items that consist entirely of whitespace characters, changing the number of whitespace characters in such an information item, or changing the number of whitespace characters at the beginning or end of any character information item.

#### 1.2.4 Use of namespaces

1. Element information items in a CellML infoset MUST belong to one of the following namespaces, unless explicitly indicated otherwise:
    1. The CellML namespace; or
    2. The MathML namespace.
2. Attribute information items in a CellML element MUST NOT be prefixed with a namespace, unless explicitly indicated otherwise.

#### 1.2.5 XML ID Attributes

1. Any element information item in the CellML namespace MAY contain an attribute with local name `id`. This attribute SHALL be treated as having attribute type ID, as defined in Section 3.3.1 of XML 1.1 [5].

### 1.3 Data representation formats in CellML

The following data representation formats are defined for use in this specification:

1. A CellML identifier:
   1. SHALL consist of a single Basic Latin alphabetical character, which MAY be followed by any combination of Basic Latin alphanumerical characters and/or Basic Latin underscores.
   2. SHALL, when comparing two identifiers, be considered identical to another identifier if and only if both identifiers are identical sequences of characters.
2. An integer string:
   1. SHALL be a base 10 representation of an integer.
   2. MAY begin with a single Basic Latin plus character U+002B or a single Basic Latin hyphen-minus character U+002D as the sign indicator.
   3. SHALL, other than the sign indicator, consist of one or more digits.
3. A basic real number string:
   1. SHALL be a base 10 representation of a real number.
   2. MAY begin with a single Basic Latin plus character U+002B or a single Basic Latin hyphen-minus character U+002D as the sign indicator.
   3. MAY contain a single decimal point separator, which SHALL be the Basic Latin full stop character U+002E.
   4. SHALL, other than the sign indicator and the decimal point separator, consist of one or more digits.
4. A real number string:
   1. SHALL be a base 10 representation of a real number $r = s \times 10^e$ where $s$ is the significand, a real number, and $e$ is the exponent, an integer.
   2. The representation of the number SHALL be the representation of the significand, optionally followed by a representation of the exponent.
   3. The significand SHALL be represented as a basic real number string.
   4. An exponent SHALL be represented by an exponent separator character, followed by the integer string representation of the value of the exponent. The exponent separator character SHALL be either the Basic Latin "E" character U+0045 or the Basic Latin "e" character U+0065.
   5. If the exponent representation is omitted, the exponent shall be zero.

CHAPTER

TWO

## ELEMENT INFORMATION ITEMS

### 2.1 The `model` element

The top-level element information item in a CellML infoset MUST be an element in the CellML namespace with a local name equal to `model`. In this specification, the top-level element is referred to as the `model` element.

1. Every `model` element MUST contain a `name` attribute.

The value of the `name` attribute MUST be a CellML identifier.

2. A `model` element MAY contain one or more additional specific element children, each of which MUST be of one of the following types:

1. A `component` element;
   2. A `connection` element;
   3. An `encapsulation` element;
   4. An `import` element; or
   5. A `units` element.

3. A `model` element MUST NOT contain more than one `encapsulation` elements.

### 2.2 The `import` element

An `import` element information item (referred to in this specification as an `import` element) is an element in the CellML namespace with a local name equal to `import`, which appears as a child of a `model` element.

1. Every `import` element MUST contain an attribute in the namespace `http://www.w3.org/1999/xlink`, with a local name equal to `href`.

The value of this attribute SHALL be a valid locator `href`, as defined in Section 5.4 of the XLink specification [6].

The `href` attribute SHALL be treated according to the XLink specification [6], by applying the rules for simple-type elements.

When describing an `import` element or one of its children, the phrase "imported CellML infoset" SHALL refer to the CellML infoset obtained by parsing the document referenced by the `href` attribute.

2. Every `import` element MAY contain one or more specific element children, each of which MUST be of one of the following types:

1. An `import component` element; or
   2. An `import units` element.

3. Any CellML infoset imported, directly or indirectly, by the imported CellML infoset MUST NOT be semantically equivalent to the importing CellML infoset (see 1.2.3.3 regarding semantic equivalence).

### 2.3 The `import units` element

An `import units` element information item (referred to in this specification as an `import units` element) is an element in the CellML namespace with a local name equal to `units`, which appears as a child of an `import` element.

1. Every `import units` element MUST contain a `name` attribute.

The value of the `name` attribute MUST be a CellML identifier.

The value of the `name` attribute MUST NOT be identical to the value of the `name` attribute of any other `units` or `import units` element in the CellML infoset.

2. Every `import units` element MUST contain a `units_ref` attribute.

The value of the `units_ref` attribute MUST be a CellML identifier.

The value of the `units_ref` attribute MUST be identical to the value of the `name` attribute on a `units` or `import units` element in the imported CellML infoset.

### 2.4 The `import component` element

An `import component` element information item (referred to in this specification as an `import component` element) is an element in the CellML namespace with a local name equal to `component`, which appears as a child of an `import` element.

1. Every `import component` element MUST contain a `name` attribute.

The value of the `name` attribute MUST be a CellML identifier.

The value of the `name` attribute MUST NOT be identical to the value of the `name` attribute of any other `component` or `import component` element in the CellML infoset.

2. Every `import component` element MUST contain a `component_ref` attribute.

The value of the `component_ref` attribute MUST be a CellML identifier.

The value of the `component_ref` attribute MUST be identical to the value of the `name` attribute on a `component` or `import component` element in the imported CellML infoset.

### 2.5 The units element

A units element information item (referred to in this specification as a units element) is an element in the CellML namespace with a local name equal to units, which appears as a child of a model element.

1. Every units element MUST contain a name attribute.

The value of the name attribute MUST be a CellML identifier.

The value of the name attribute MUST NOT be identical to the value of the name attribute of any other units element or import units element in the CellML infoset.

2. The value of the name attribute MUST NOT be identical to the name of any of the units listed in Table 3.1: Built-in units (see 3.2 Units references).

3. A units element MAY contain one or more unit element children.

### 2.6 The `unit` element

A `unit` element information item (referred to in this specification as a `unit` element) is an element in the CellML namespace with a local name equal to `unit`, which appears as a child of a `units` element.

1. Every `unit` element MUST contain a `units` attribute.

The value of the `units` attribute MUST be a valid units reference, as defined in 3.2 Units references.

1. For the purpose of the constraint in the next paragraph, the `units` element inclusion digraph SHALL be defined as a conceptual digraph which SHALL contain one node for every `units` element in the CellML model.

The `units` element inclusion digraph SHALL contain an arc from `units` element *A* to `units` element *B* if and only if `units` element *A* contains a `unit` element with a `units` attribute value that is identical to the `name` attribute value of `units` element *B*.

2. The element inclusion digraph MUST NOT contain any cycles.

2. A `unit` element MAY contain any of the following attributes:

1. The `prefix` attribute. If present, the value of the attribute MUST meet the constraints specified in 3.3 Interpretation of `units` elements.

2. The `multiplier` attribute. If present, the value of the attribute MUST be a real number string.

3. The `exponent` attribute. If present, the value of the attribute MUST be a real number string.

### 2.7 The `component` element

A `component` element information item (referred to in this specification as a `component` element) is an element in the CellML namespace with a local name equal to `component`, which appears as a child of a `model` element.

1. Every `component` element MUST contain a `name` attribute.

The value of the `name` attribute MUST be a CellML identifier.

The value of the `name` attribute MUST NOT be identical to the value of the `name` attribute on any other `component` element or `import component` element in the CellML infoset.

2. A `component` element MAY contain one or more specific element children, each of which MUST be of one of the following types:

1. A `math` element;
   2. A `reset` element; or
   3. A `variable` element.

### 2.8 The `variable` element

A `variable` element information item (referred to in this specification as a `variable` element) is an element in the CellML namespace with a local name equal to `variable`, which appears as a child of a `component` element.

1. Every `variable` element MUST have exactly one of each of the following attributes:
   1. The `name` attribute. The value of the `name` attribute MUST be a CellML identifier.

The value of the `name` attribute MUST NOT be identical to the value of the `name` attribute on any sibling `variable` element.
   2. The `units` attribute. The value of the `units` attribute MUST be a valid units reference, as defined in 3.2 Units references.
2. Every `variable` element MAY contain one or more of the following attributes:
   1. The `interface` attribute. If the attribute is present, it MUST have value of `public`, `private`, `public_and_private`, or `none`.
   2. The `initial_value` attribute. If the attribute is present, it MUST meet the requirements described by 3.6 Interpretation of `initial_value` attributes.

### 2.9 The `reset` element

A `reset` element information item (referred to in this specification as a `reset` element) is an element in the CellML namespace with a local name equal to `reset`, which appears as a child of a `component` element.

1. Every `reset` element MUST have exactly one of each of the following attributes:
   1. The `variable` attribute. The value of the `variable` attribute MUST be a valid variable reference, as defined in 3.5 Variable references.
   2. The `test_variable` attribute. The value of the `test_variable` attribute MUST be a valid variable reference, as defined in 3.5 Variable references.
   3. The `order` attribute. The value of the `order` attribute MUST be an integer string.

The value of the `order` attribute MUST be unique for all `reset` elements with `variable` attributes that reference variables in the same equivalent variable set (see 3.10 Interpretation of `map_variables` elements).
2. A `reset` element MUST contain exactly two element children, which MUST be one of each of the following types:
   1. A `reset_value` element; and
   2. A `test_value` element.

### 2.10 The `test_value` element

A `test_value` element information item (referred to in this specification as a `test_value` element) is an element in the CellML namespace with a local name equal to `test_value`, which appears as a child of a `reset` element.

1. A `test_value` element MUST contain exactly one `math` element child.

### 2.11 The `reset_value` element

A `reset_value` element information item (referred to in this specification as a `reset_value` element) is an element in the CellML namespace with a local name equal to `reset_value`, which appears as a child of a `reset` element.

1. A `reset_value` element MUST contain exactly one `math` element child.

### 2.12 The math element

A math element information item (referred to in this specification as a math element) is an element in the MathML namespace, which appears as a child of a component element, a test_value element, or a reset_value element.

1. A math element MUST be the top-level element of a Content MathML tree, as described in MathML 2.0 [7].
2. Each element child of a math element MUST have an element-type name that is listed in Table 2.1: The math element.
3. The contents of a MathML ci element MUST be a valid variable reference, as defined in 3.5 Variable references.
4. A MathML cn element MUST have an attribute in the CellML namespace, with a local name equal to units.

The value of the units attribute MUST be a valid units reference, as defined in 3.2 Units references.
5. A cn element MUST be base 10, and MUST be of the following types: real or e-notation.

Table 2.1: Supported MathML elements

| Element Category | Element List |
|---|---|
| Simple Operands | ci, cn, sep |
| Basic Structural | apply, piecewise, piece, otherwise |
| Relational and Logical Operators | eq, neq, gt, lt, geq,<br>leq, and, or, xor, not |
| Arithmetic Operators | plus, minus, times, divide,<br>power, root, abs,<br>exp, ln, log,<br>floor, ceiling,<br>min, max, rem |
| Calculus Elements | diff |
| Qualifier Elements | bvar, logbase, degree * |
| Trigonometric Operators | sin, cos, tan, sec, csc, cot,<br>sinh, cosh, tanh, sech, csch, coth,<br>arcsin, arccos, arctan,<br>arcsec, arccsc, arccot,<br>arcsinh, arccosh, arctanh,<br>arcsech, arccsch, arccoth |
| Mathematical and Logical Constants | pi, exponentiale, notanumber,<br>infinity, true, false |

* degree MUST be a child of root or diff

### 2.13 The `encapsulation` element

An `encapsulation` element information item (referred to in this specification as an `encapsulation` element) is an element in the CellML namespace with a local name equal to `encapsulation`, which appears as a child of a `model` element.

1. An `encapsulation` element MAY contain one or more `component_ref` element children.

### 2.14 The `component_ref` element

A `component_ref` element information item (referred to in this specification as a `component_ref` element) is an element in the CellML namespace with a local name equal to `component_ref`, which appears as a child of an `encapsulation` element.

1. Every `component_ref` element MUST contain a `component` attribute.

The value of the `component` attribute MUST be a valid component reference, as defined in 3.4 Component references.

The value of the `component` attribute MUST NOT be identical to the value of the `component` attribute on any other `component_ref` element in the CellML infoset.

2. Every `component_ref` element MAY in turn contain one or more `component_ref` element children.

### 2.15 The `connection` element

A `connection` element information item (referred to in this specification as a `connection` element) is an element in the CellML namespace with a local name equal to `connection`, which appears as a child of a `model` element.

1. Each `connection` element MUST contain a `component_1` attribute.

The value of the `component_1` attribute MUST be a valid component reference, as defined in 3.4 Component references.

2. Each `connection` element MUST contain a `component_2` attribute.

The value of the `component_2` attribute MUST be a valid component reference, as defined in 3.4 Component references.

3. The value of the `component_1` attribute MUST NOT be identical to the value of the `component_2` attribute.

4. A CellML infoset MUST NOT contain more than one `connection` element with a given pair of components referenced by the `component_1` and `component_2` attribute values, in any order.

5. A `connection` element MAY contain one or more `map_variables` element children.

### 2.16 The map_variables element

A map_variables element information item (referred to in this specification as a map_variables element) is an element in the CellML namespace with a local name equal to map_variables, which appears as a child of a connection element.

1. Each map_variables element MUST contain a variable_1 attribute.

The value of the variable_1 attribute MUST be a valid variable reference, as defined in 3.5 Variable references.

2. Each map_variables element MUST contain a variable_2 attribute.

The value of the variable_2 attribute MUST be a valid variable reference, as defined in 3.5 Variable references.

3. A connection element MUST NOT contain more than one map_variables element with a given variable_1 attribute value and variable_2 attribute value pair.

CHAPTER

THREE

## INTERPRETATION

### 3.1 Interpretation of `import` elements

1. Each `import units` or `import component` element present in a CellML infoset (the importing infoset) SHALL define a new and distinct instance of the CellML infoset (the imported infoset) that is specified by the parent `import` element's `href` attribute.
2. A units reference occurring within an imported element, SHALL be resolved with respect to the imported infoset.
3. When determining the equivalent variable set of a variable in an imported component:
    1. Connections defined in the importing infoset SHALL be handled as described in 3.10 Interpretation of `map_variables` elements.
    2. Connections defined in the imported infoset SHALL be handled as follows:
        1. Connections to components in the encapsulated set of the imported component SHALL be maintained, and this rule SHALL be applied recursively; and
        2. Connections to components in the sibling set, or to the encapsulation parent of the imported component SHALL NOT be maintained.

### 3.2 Units references

A "units reference" is an attribute value that specifies the physical units a variable or number is in.

1. A units reference SHALL be a CellML identifier.
2. The units identified by a units reference SHALL be determined as follows:
    1. If the units reference is identical to a value in the "Name" column of Table 3.1: Built-in units, then it SHALL refer to the built-in units from the same row of the table.
    2. If the units reference is identical to the value of the `name` attribute of a `units` element in the same infoset, then it SHALL refer to the units specified by that element.
    3. If the units reference is identical to the value of the `name` attribute of an `import units` element in the same infoset, then it SHALL refer to units from the infoset defined by the `import units` element (see 3.1 Interpretation of `import` elements`). The units specified SHALL then be determined by treating the value of the `units_ref` attribute on the `import units` element as a units reference within the imported infoset. If necessary, this rule SHALL be applied recursively.
3. If no units can be identified using the rules above, the attribute value SHALL NOT be a valid units reference.

Table 3.1: Built-in units

| **Name** | **Unit reduction tuple**<br>multiplier·(base, exponent) |
|---|---|
| `ampere` | - |
| `becquerel` | (`second`, -1) |
| `candela` | - |
| `coulomb` | (`second`, 1), (`ampere`, 1) |
| `dimensionless` | - |
| `farad` | (`kilogram`, -1), (`metre`, -2), (`second`, 4), (`ampere`, 2) |
| `gram` | 0.001·(`kilogram`, 1) |
| `gray` | (`metre`, 2), (`second`, -2) |
| `henry` | (`kilogram`, 1), (`metre`, 2), (`second`, -2), (`ampere`, -2) |
| `hertz` | (`second`, -1) |
| `joule` | (`kilogram`, 1), (`metre`, 2), (`second`, -2) |
| `katal` | (`second`, -1), (`mole`, 1) |
| `kelvin` | - |
| `kilogram` | - |
| `litre` | 0.001·(`metre`, 3) |
| `lumen` | (`candela`, 1) |
| `lux` | (`metre`, -2), (`candela`, 1) |
| `metre` | - |
| `mole` | - |
| `newton` | (`kilogram`, 1), (`metre`, 1), (`second`, -2) |
| `ohm` | (`kilogram`, 1), (`metre`, 2), (`second`, -3), (`ampere`, -2) |
| `pascal` | (`kilogram`, 1), (`metre`, -1), (`second`, -2) |
| `radian` | (`dimensionless`, 1) |
| `second` | - |
| `siemens` | (`kilogram`, -1), (`metre`, -2), (`second`, 3), (`ampere`, 2) |
| `sievert` | (`metre`, 2), (`second`, -2) |
| `steradian` | (`dimensionless`, 1) |
| `tesla` | (`kilogram`, 1), (`second`, -2), (`ampere`, -1) |
| `volt` | (`kilogram`, 1), (`metre`, 2), (`second`, -3), (`ampere`, -1) |
| `watt` | (`kilogram`, 1), (`metre`, 2), (`second`, -3) |
| `weber` | (`kilogram`, 1), (`metre`, 2), (`second`, -2), (`ampere`, -1) |

### 3.3 Interpretation of `units` elements

1. The `units` element SHALL be interpreted as the product of its `unit` element children, according to the following rules:

1. The prefix term is a conceptual property of `unit` elements. If the `unit` element does not have a `prefix` attribute, the prefix term SHALL have value 0. If the `prefix` attribute has a value which is an integer string, then the value of the prefix term SHALL be the numerical value of that string. Otherwise, the `prefix` attribute MUST have a value taken from the “Name” column of Table 3.2: Interpretation of `units` elements, and the prefix term SHALL have the value taken from the “Value” column of the same row.

2. The exponent term is a conceptual property of `unit` elements. If a `unit` element has no `exponent` attribute, the exponent term SHALL have value 1.0. Otherwise, the value of the `exponent` attribute MUST be a real number string, and the value of the exponent term SHALL be the numerical value of that string.

3. The multiplier term is a conceptual property of `unit` elements. If a `unit` element has no `multiplier` attribute, the multiplier term SHALL have value 1.0. Otherwise, the value of the `multiplier` attribute MUST be a real number string, and the value of the multiplier term SHALL be the numerical value of that string.

4. The relationship between the product, $P$, of numerical values given in each and every child `unit` element’s `units` attribute, to a numerical value, $x$, with units given by the encompassing `units` element, SHALL be

$$x[u_x] = \frac{1}{m_1 \cdots m_n (10^{p_1})^{e_1} \cdots (10^{p_1})^{e_n}} \left[ \frac{u_x}{u^{e_1} \cdots u^{e_n}} \right] P \left[ u^{e_1} \cdots u^{e_n} \right]$$

where $u_x$ denotes the units of the `units` element; $p_i$, $e_i$, $m_i$ and $u_i$ refer to the prefix, exponent and multiplier terms and units of the $i^{th}$ `unit` child element, respectively. Square brackets encompass the units of numerical values.

2. For the purposes of this specification, the “irreducible units” of a model SHALL consist of:

1. The units defined in a model that are not defined in terms of other units (i.e. the set of `units` elements in the CellML model which have no `unit` child elements).

2. The built-in irreducible units (those built-in units with “-” in the “Unit reduction tuple” column of Table 3.1: Built-in units) referenced by variables or other units in the model.

3. The “unit reduction” is a conceptual property of `units` elements. It consists of a set of tuples where each tuple is composed of a “unit name” and a real-valued “exponent”. The set of tuples SHALL be determined as follows:

1. If the `units` element has no `unit` child elements, then the set of tuples SHALL have a single member, which SHALL consist of the name of the `units` element and the exponent 1.0.

2. If the `units` element has one or more `unit` child elements, then the set of tuples SHALL consist of the entire collection of tuples given by all `unit` child elements.

Tuples for each `unit` child element SHALL be determined as follows:

1. If the units reference of the `unit` child element is to a single unit which is an irreducible unit, then the set of tuples SHALL have a single member, which SHALL consist of the name of the irreducible unit being referenced and the exponent 1.0.

2. If the units reference of the `unit` child element is to built-in units other than an irreducible unit, then the tuples SHALL be derived directly from Table 3.1: Built-in units. Specifically, the set of tuples SHALL consist of the tuples given in the “Unit reduction tuple” column of the row for which the value in the “Name” column is identical to the name of the units reference.

3. If the units reference of the `unit` child element is to a unit which is neither built-in, nor an irreducible unit, then the set of tuples SHALL be defined recursively as the set of tuples for the `units` element so referenced.

4. The exponents of each tuple in the set for the current `unit` element, as derived by following rules 3.3.3.2.1, 3.3.3.2.2 or 3.3.3.2.3 above, SHALL be multiplied by the exponent term of the current, referencing, `unit` element.

3. Tuples which have the unit name of “dimensionless” SHALL be removed from the set of tuples. Note that this can result in the set of tuples being empty.

4. If the set of tuples contains tuples which have the same unit name, then those tuples SHALL be combined into a single tuple with that unit name and an exponent being the sum of those tuples’ exponents. If the resulting tuple’s exponent is zero, then the tuple SHALL be removed from the set of tuples. Note that this can result in the set of tuples being empty.

Table 3.2: Prefix values

| Name | Value |
|---|---|
| `yotta` | 24 |
| `zetta` | 21 |
| `exa` | 18 |
| `peta` | 15 |
| `tera` | 12 |
| `giga` | 9 |
| `mega` | 6 |
| `kilo` | 3 |
| `hecto` | 2 |
| `deca` | 1 |
| `deci` | −1 |
| `centi` | −2 |
| `milli` | −3 |
| `micro` | −6 |
| `nano` | −9 |
| `pico` | −12 |
| `femto` | −15 |
| `atto` | −18 |
| `zepto` | −21 |
| `yocto` | −24 |

### 3.4 Component references

A "component reference" is an attribute value that specifies a CellML component.

1. A component reference SHALL be a CellML identifier.
2. The component identified by a component reference SHALL be determined as follows:
   1. If the component reference is identical to the value of the `name` attribute of a `component` element in the same infoset, then it SHALL refer to the component specified by that element.
   2. If the component reference is identical to the value of the `name` attribute of an `import component` element in the same infoset, then it SHALL refer to a component from the infoset defined by the `import component` element (see 3.1 Interpretation of `import` elements). The component specified SHALL then be determined by treating the value of the `component_ref` attribute on the `import component` element as a component reference within the imported infoset. If necessary, this rule SHALL be applied recursively.
3. If no component can be identified using the rules above, the attribute value SHALL NOT be a valid component reference.

### 3.5 Variable references

A "variable reference" is an attribute value that specifies a CellML variable.

1. A variable reference SHALL be a CellML identifier.
2. The variable identified by a variable reference SHALL be determined as follows:
    1. When present in a descendant of a `component` element, it SHALL refer to the variable of the same name within that component.
    2. When present in the `variable_1` attribute of a `map_variables` element, it SHALL refer to the variable of the same name in the component identified by the `component_1` attribute in the same `map_variables` element.
    3. When present in the `variable_2` attribute of a `map_variables` element, it SHALL refer to the variable of the same name in the component identified by the `component_2` attribute in the same `map_variables` element.
3. If no variable can be identified using the rules above, the attribute value SHALL NOT be a valid variable reference.

### 3.6 Interpretation of `initial_value` attributes

1. The `initial_value` attribute of a `variable` element MUST either be a real number string, or a variable reference.
2. The conditions under which initial values hold are (by design) not defined in a CellML model.
3. Where the `initial_value` attribute is a real number string, it SHALL be interpreted as a statement that the variable on which the attribute appears is equal to that real number value when the initial values hold.
4. Where the `initial_value` attribute is a variable reference, it SHALL be interpreted as a statement that the variable on which the attribute appears is equal to the referenced variable when the initial values hold.

### 3.7 Effect of `units` on a `variable`

1. The target of the `units` attribute on a variable is referred to as the "variable units", and the corresponding unit reduction (see 3.3 Interpretation of `units` elements) is referred to as the "variable unit reduction".

### 3.8 Interpretation of `math` elements

1. The following `component` elements SHALL, for the purposes of this specification, be "pertinent component elements":
    1. All `component` elements in the top-level CellML infoset for the CellML model;
    2. All `component` elements referenced by `import component` elements (see 3.1 Interpretation of `import` elements) in the top-level CellML infoset; and
    3. All `component` elements which are descendants in the encapsulation digraph (see 3.9 Interpretation of `encapsulation` elements) of a pertinent `component` element.
2. Every MathML element in the CellML model which appears as a direct child of a MathML `math` element, which in turn appears as a child of a pertinent `component` element, SHALL be treated, in terms of the semantics of the mathematical model, as a statement which holds true unconditionally.
3. Units referenced by a `units` attribute SHALL NOT affect the mathematical interpretation of the CellML model.

### 3.9 Interpretation of `encapsulation` elements

1. For the purposes of this specification, there SHALL be a conceptual “encapsulation digraph” in which there is exactly one node for every component in the CellML model.
2. Where a `component_ref` element appears as a child of another `component_ref` element, there SHALL be an arc in the encapsulation digraph, and that arc SHALL be from the node corresponding to the component referenced by the parent `component_ref` element, and to the node corresponding to the component referenced by the child `component_ref` element.
3. The “encapsulated set” for a component $A$ SHALL be the set of all components $B$ such that there exists an arc in the encapsulation digraph from the node corresponding to $A$ to the node corresponding to $B$.
4. The “encapsulation parent” for a component $A$ SHALL be the component corresponding to the node which is the parent node in the encapsulation digraph of the node corresponding to $A$. A component SHALL NOT appear as child of more than one encapsulation parent.
5. The “sibling set” for a component $A$ SHALL be the set of all components which have the same encapsulation parent as $A$, or in the case that $A$ has no encapsulation parent, SHALL be the set of all components which do not have an encapsulation parent.
6. The “hidden set” for a component $A$ SHALL be the set of all components $B$ where component $B$ is not in the encapsulated set for component $A$, and component $B$ is not the encapsulation parent of component $A$, and component $B$ is not in the sibling set for component $A$.

### 3.10 Interpretation of `map_variables` elements

1. For the purposes of this specification, the conceptual "variable equivalence network" SHALL be an undirected graph with one node for every `variable` element in the CellML model. The arcs of this graph SHALL be equivalences defined in the CellML model.
2. For each `map_variables` element present in the CellML model, we define variables *A* and *B* for use in the rules in this section as follows:
   1. Variable *A* SHALL be the variable referenced by the encompassing `connection` element's `component_1` and this `map_variables` element's `variable_1` attribute; and
   2. Variable *B* SHALL be the variable referenced by the encompassing `connection` element's `component_2` and this `map_variables` element's `variable_2` attribute.
3. For every `map_variables` element present in the CellML model, there SHALL be an arc in the variable equivalence network.
   1. One endpoint of the arc in the variable equivalence network SHALL be the node corresponding to variable *A*; and
   2. One endpoint of the arc in the variable equivalence network SHALL be the node corresponding to variable *B*.
4. CellML models MUST NOT contain any pair of `map_variables` elements which duplicates an existing arc in the variable equivalence network.
5. The variable equivalence network MUST NOT contain any cycles.
6. For a given variable, the "available interfaces" SHALL be determined by the value of the `interface` attribute on the corresponding `variable` element as follows:
   1. A value of `public` specifies that the variable has a public interface;
   2. A value of `private` specifies that the variable has a private interface;
   3. A value of `public_and_private` specifies that the variable has both a public and a private interface;
   4. A value of `none` specifies that the variable has no interface; or
   5. If the `interface` attribute is absent, then the variable has no interface.
7. The "applicable interfaces" for variables *A* and *B* in components *AA* and *BB* respectively SHALL be defined as follows:
   1. When component *AA* is in the sibling set of component *BB* (and vice versa), the applicable interface for both variables *A* and *B* SHALL be the public interface.
   2. When component *AA* is in the encapsulated set of component *BB*, the applicable interface for variable *A* SHALL be the public interface, and the applicable interface for variable *B* SHALL be the private interface.
   3. When component *BB* is in the encapsulated set of component *AA*, the applicable interface for variable *B* SHALL be the public interface, and the applicable interface for variable *A* SHALL be the private interface.

It is noted, for the avoidance of doubt, that if components *AA* and *BB* are in each other's hidden set, there are no applicable interfaces for the variables *A* and *B*.
8. CellML models MUST only contain `map_variables` elements where the applicable interfaces for both variables are available.
9. For each `map_variables` element present in the CellML model, the variable unit reduction (see 3.7 Effect of `units` on a `variable`) of variable *A* MUST have an identical set of tuples to the variable unit reduction of variable *B*. Two sets of tuples SHALL be considered identical if all of the tuples from each set are present in the other, or if both sets are empty. Two tuples are considered identical if and only if both the unit name and exponent of each tuple are identical.
10. Tuples differing by a multiplying factor in their unit reduction MUST be taken into account when interpreting the numerical values of the variables (see 3.3 Interpretation of `units` elements).

11. The `variable` elements in a CellML model SHALL be treated as belonging to a single "equivalent variable set". Each set of equivalent variables is the set of all `variable` elements for which the corresponding nodes in the variable equivalence network form a connected subgraph. Each set of equivalent variables represents one variable in the underlying mathematical model.

### 3.11 Interpretation of `reset` elements

1. For the purposes of this section, we define the "reset variable" to be the variable referenced by a `reset` element's `variable` attribute, and the "test variable" to be the variable referenced by its `test_variable` attribute.
2. Each `reset` element describes a change to be applied to the reset variable when specified conditions are met during the simulation of the model.
3. All `reset` elements SHALL be considered sequentially for the equivalent variable set (see 3.10 Interpretation of `map_variables` elements) to which the reset variable belongs. The sequence SHALL be determined by the value of the reset element's `order` attribute, lowest (least positive / most negative) having priority.
4. The condition under which a reset occurs SHALL be defined by the equality of evaluation of the test variable and the evaluation of the MathML expression encoded in the `test_value`.
5. When a reset occurs, the reset variable SHALL be set to the result of evaluating the MathML expression encoded in the `reset_value`.

CHAPTER

FOUR
